# Diel pattern of corticosterone metabolites in Arctic barnacle goslings (*Branta leucopsis*) under continuous natural light

**DOI:** 10.1371/journal.pone.0182861

**Published:** 2017-08-07

**Authors:** Isabella B. R. Scheiber, Margje E. de Jong, Jan Komdeur, Elisabeth Pschernig, Maarten J. J. E. Loonen, Eva Millesi, Brigitte M. Weiß

**Affiliations:** 1 Behavioural and Physiological Ecology, Groningen Institute for Evolutionary Life Sciences, The University of Groningen, Groningen, The Netherlands; 2 Arctic Centre, The University of Groningen, Groningen, The Netherlands; 3 Department of Behavioural Biology, University of Vienna, Vienna, Austria; 4 Behavioural Ecology Research Group, University of Leipzig, Germany; 5 Max Planck Institute for Evolutionary Anthropology, Leipzig, Germany; McGill University, CANADA

## Abstract

Here we describe the excretion pattern of corticosterone metabolites collected from droppings in barnacle goslings (*Branta leucopsis*) raised under 24 hours of continuous natural light in the Arctic. In lower latitudes, circulating corticosterone peaks around waking and shows a nadir between midnight and 4:00, whereas the peak and nadir are time-delayed slightly when measuring corticosterone metabolites from droppings. Photoperiod, along with other environmental factors, helps to entrain an animal’s endogenous rhythm to that of the natural world. North of the Arctic Circle, photoperiod may not be a reliable cue as light is continuously absent during the winter and continuously present during the summer. Here, for the first time, we used droppings to describe a 24-hour excretion pattern of corticosterone metabolites (CORTm). By applying circular statistics for dependent data, we found a diel rhythmic pattern even under continuous natural light. We discuss potential alternative ‘Zeitgeber’ that may function even in the polar regions, focusing on melatonin. We propose a line of research to measure melatonin non-invasively from droppings. We also provide a validation of the adopted enzyme immunoassay (EIA) that was originally developed for greylag geese.

## Introduction

The 24-hour rotation of the Earth subjects most living organisms to predictable daily rhythms of light intensity and temperature [1]. Endogenous biological clocks coordinate and phase biochemical, physiological and behavioural processes, which occur rhythmically and are restricted to specific times of the day or season [1, 2].

From an evolutionary perspective, the molecular basis of circadian clocks and clock function is highly conserved throughout the vertebrates. Circadian clocks are generally induced by transcriptional-translational feedback loops through rhythmic expression of clock gene products, which, in turn, switch on other clock genes in approximately 24-hour cycles. Synchronization of endogenous circadian network systems is acquired through one or several endogenous ‘Master’ clocks, which then coordinate more peripheral clocks. In birds, several dissociated but interacting ‘Master’ clocks are located in the retina, the pineal organ, and suprachiasmatic nuclei (SCN, [1]). Circadian clocks deviate slightly from a 24-hour rhythm, and therefore must be entrained by external stimuli, so-called ‘*Zeitgeber*’, to ensure synchronization with real environmental time. Photoperiod provides the most important Zeitgeber in many species [3].

The physiological basis by which animals assess environmental day length is governed by the rhythmic excretion of hormones [1, 3]. Although hormone excretion, in part, has a genetic basis, rhythmicity is frequently maintained with photoperiod as a Zeitgeber. Melatonin, a hormone secreted primarily by the pineal gland and responsible for modulating sleep patterns, is a crucial product of circadian master clocks. It provides the hormonal signal transducing day length for peripheral clocks and daily physiological processes [1, 3]. Generally, melatonin is secreted in a diel pattern, with an extended peak occurring at night, and basal secretion during the day. The duration of the nightly pineal melatonin secretion is inversely related to day length and its secretion drives enduring changes in many physiological systems, including the hypothalamic-pituitary-adrenal stress axis (HPA, [3]), one of the two central stress response systems in vertebrates. HPA activation ultimately results in the release of glucocorticoids (GCs, *e*.*g*. cortisol in mammals, corticosterone in birds).

GCs are a group of adrenal cortical steroids that are, for example, involved in fat metabolism [4], frequently in carbohydrate metabolism (but see [5, 6]), suppression of immune function and stress responses [2]. They are well known for their robust rhythmicity in circulating levels [7–10] as well as for their involvement in coordinating peripheral clocks [11]. Both seasonal (*e*.*g*. [12–15]) and diel variation ([16, 17], but see [15]) of GCs exists, with daily peaks occurring just before or right around waking, whereas a nadir occurs around midnight to 4:00 [2, 18] in latitudes with year-round day-night cycles.

While at lower latitudes photoperiod provides a reliable cue for changes in hormone levels, it becomes less dependable towards the equator, where daylength remains more or less constant over the course of the year. Above the Arctic and below the Antarctic circle (66°N and S), where natural light is completely absent in winter but continuously present during the summer, photoperiod also may provide an undependable signal [9] and potentially challenge the accurate function of diel rhythms [19]. However, despite the lack of photoperiodicity, other potential ‘Zeitgeber’, such as diurnal changes in light intensity, polarization patterns, solar azimuth, UV radiation, changes in the spectral composition of light or ambient temperature are present ([19–22], see [1, 23] for recent reviews).

Studies on circadian rhythmicity in polar regions have focussed on both behavioural patterns and physiological parameters that are controlled by circadian clocks (see [1, 23] for detailed reviews). Regarding behavioural activity patterns, there is large variation in activity patterns of Arctic breeding vertebrates, with some species displaying either (i) entrained 24-hour activity cycles, (ii) arrhythmic or (iii) completely free-running cycles in the Arctic summer [24]. In herbivores, for example, being continuously active is a common phenomenon, even at low latitudes (see [23] for a recent review). Rhythmicity, however, is plastic and may change seasonally, driven by annual changes of day length [24–26]. Svalbard rock ptarmigan (*Lagopus muta hyperborea*), the only permanent resident avian species on Svalbard, for example, show ultradian activity patterns and feeding occurs periodically during polar summer and winter days, whereas during spring and fall, activity patterns are diurnal and feeding occurs mainly during daylight hours [25]. Similarly, phytophagous barnacle geese (*Branta leucopsis*) breeding on Svalbard also maintain an ultradian cyclic activity pattern of approximately 1.5 hours of feeding, followed by a half hour rest in early July, whereas in August a midnight sleeping interval of four hours is interspersed (M. J. J. E. Loonen, pers. obs.). These findings support ideas by Bloch *et al*. [23], who suggested that ultradian activity patterns in polar regions are linked with feeding behaviour and digestive processes, particularly in herbivores.

With respect to physiological parameters in polar regions, studies have focussed mainly on rhythmicity of hormones. Whereas under artificial constant light pineal function is hampered and results in a suppression of melatonin excretion, several Arctic and Antarctic species, such as willow warblers **(***Phylloscopus trochilus***),** Lapland longspurs (*Calcarius lapponicus*) or Adélie penguins (*Pygoscelis adeliae*) maintain clear diel melatonin rhythmicity during polar summer days [19, 21, 22, 27, 28]. Other studies, however, found no such pattern (emperor penguin (*Aptenodytes forsteri*), [29], Svalbard ptarmigan, [30]). Along the same lines, there are multiple studies that have shown that several species of migratory birds still modulate circulating levels of corticosterone in response to a capture–stress protocol (*e*.*g*. [12, 13, 31]) even under 24 hours of continuous natural daylight, whereas in other species the daily rhythm components of plasma corticosterone concentrations were absent (Adélie penguin, [9], common eider (*Somateria mollissima*), [32]). In the latter study, the absence of a clear diel corticosterone pattern in common eiders was linked to the influence of corticosterone on constant foraging activity [32].

Only very few studies are available that specifically were aimed at determining baseline levels of corticosterone over the course of a full day [9, 33, 34]. The detection of short peaks of corticosterone metabolites entails a frequent sample collection [35], and as recently pointed out by Goymann & Trappschuh [33], the best sampling protocol for describing diel patterns of hormone metabolites from faeces would be to collect all droppings in specific sampling intervals during a single 24-hour period. This, however, may cause disturbance for the animals [33] and is time-consuming, particularly in a natural setting, as animals need to be followed constantly for 24 hours. Therefore, most studies sampled only snapshots by either collecting droppings at specific points in time over the course of 24-hours (*e*.*g*. [34, 36, 37]) or in certain intervals over several days until a 24-hour circle was completed (*e*.*g*. 2 hours/ day, the next 2 hours on the following day, and so on; [33]). For prospective studies on stress coping abilities in wild barnacle geese under a complete day of natural light it was essential to describe the detailed excretion pattern of corticosterone metabolites. Collecting all droppings of specific individuals, however, bears the cost of smaller sample size due to the high efforts involved, and potentially causing long-lasting disturbance. The latter problem can be solved by using species-appropriately human-raised animals that do not shy away from humans while displaying natural behaviour [38]. Utilizing free-ranging barnacle goslings (*Branta leucopsis*) hand-raised under natural light conditions gave us the unique opportunity to describe the diel excretion pattern of corticosterone metabolites over the course of 24 hours of continuous natural daylight. We predicted the absence of a robust diel rhythm of corticosterone excretion in herbivorous barnacle geese due to their ultradian foraging activity during the Arctic summer.

### Validation of an enzyme immunoassay for the measurement of faecal glucocorticoid metabolites in barnacle geese

In many studies, circulating corticosterone levels are measured from plasma by drawing blood. However, capture and the sampling procedure elicit a stress response, which results in an increase of GCs within minutes (*e*.*g*. [12, 39, 40]) and may pose a problem when interested in baseline levels of GCs. A suitable alternative to blood sampling is the non-invasive determination of GC metabolites from droppings [41, 42]. One advantage of measuring GCs from droppings is that successive samples can readily be collected without having to be concerned about GC levels first having to return to baseline levels before the next sample provides a reliable measure. Additionally, GC metabolites from droppings provide a collective measure of circulating hormone concentrations prior to defecation, whereas GCs determined from plasma reflect circulating levels at exactly the point in time of collection ([40], for recent reviews see [42, 43]).

The method of analysing hormone metabolites from droppings is well established [43]. As hormone metabolites rather than the actual circulating hormone itself are determined in droppings, the method always requires a validation of the potential enzyme immunoassay (EIA), before it can be applied reliably to a given species [40, 44–47] as it may not detect relevant GCs even in closely related species [48, 49]. Immunoassays react to metabolites from all age groups if their nutritional strategies, *i*.*e*. the dietary preference, gastrointestinal anatomy, digestive physiology, biochemical capabilities, and commensal microflora [50], are similar. Thus, once validated, the same assay can be used for adults and juveniles [51–53]. Our second aim, therefore, was to establish the validity of one available EIA through a capture and restraint challenge performed on a flock of captive barnacle geese. This assay has been applied successfully in a variety of studies in greylag geese (*Anser anser*) [54–57].

## Materials and methods

### Ethical statement

The study employed hand-raised goslings and complied with all current Norwegian laws and regulations concerning work with wildlife (FOTS: 5468 of the Norwegian Animal Research Authority to M. J. J. E. Loonen). Sample collection in captive geese was performed under DEC License 6778 (to I. B. R. Scheiber, Groningen Institute of Evolutionary Life Sciences, Groningen, The Netherlands). No other manipulations of the geese, which would have required additional licenses, were performed.

### Biological validation of the corticosterone enzyme immunoassay

#### Study population and sample collection

Data collection for the biological validation was performed in the goose pens (length x width: 68 m x 60 m) of the animal care facility at the University of Groningen (53°14’N, 6°32’E) by M. E. de Jong and I. B. R. Scheiber. Details of the facilities are described elsewhere [57]. At the time of data collection, *i*.*e*. March 17^th^ and 18^th^ 2015, the captive mixed species flock consisted of 26 adult greylag and 29 adult barnacle geese, which could all be identified individually by a unique combination of coloured leg bands. For the validation we collected repeated droppings from 24 barnacle geese (13 males and 11 females).

On both days, data collection started well after the early morning corticosterone peak [58] under similar weather conditions [54]. Temperatures at the start and end of the collection days were: 8°C at 08:45 and 15°C at 16:00 on March 17^th^, and 9°C at 08:45 and 13°C at 16:25 on March 18^th^, respectively.

The data collection was split into a control day and a challenge day. On the control day (March 17^th^), geese were fed without any disturbance by spreading their regular amount of food over the ground in the goose pen, as is done on a daily basis year round. From 09:00 to 15:30 we collected all droppings (*n* = 78 samples) whenever we saw a goose defecate and the sample could be assigned to an individual.

High social density [55] and confinement [59] are considered serious stressors for geese. We therefore opted for a chase and subsequent confinement of the geese in a small area as the stressor for our validation experiment. On the challenge day (March 18^th^), we first chased geese into a permanently set up funnel trap (length x width: 6 m x 2.5 m) at 08:45 and then kept them confined for 30 min. Before they were released one by one, their feathers were checked for growth, and if necessary clipped to maintain flightlessness. Meanwhile, the same amount of food was spread in the pen as on the control day. On the challenge day, data were collected after the release of all geese, *i*.*e*. from 09:45 to 16:00. Again we collected all droppings that we could assign to individual geese (*n* = 72 samples). For most of the sampled geese (*n*_*total*_ = 20; 11 males and 9 females) we were able to collect droppings on both days (S1 Table). As goose droppings consist of an inseparable mixture of uric acid and faecal matter, we collected and analysed both [57, 60]. Dropping samples were frozen at -20°C within 1 hour after collection, as freezing maintains accurate hormone metabolite levels for extended periods of time [61]. Samples were shipped frozen to the Department of Behavioural Biology at the University of Vienna (Vienna, Austria) for analysis.

#### Extraction of immunoreactive corticosterone metabolites and determination of hormone metabolite levels

Defrosted samples were weighed in at 0.5 g of wet faeces, as either of the two commonly used extraction methods (wet feces/methanol or dried feces/ethanol) were proposed to give similar results [45, 60]. We crushed and homogenised samples and suspended 0.5 g of each sample in 4.5 ml of 60% methanol for extraction. Extracting faeces with a lower proof percentage of alcohol, *i*.*e*. 60% rather than 80% proof, is recommended for birds from which faeces and urine are excreted together [45, 60]. Samples were vortexed at 1,500 rpm for 30 min, and centrifuged (2,796 g) for 15 min. After that the supernatant was transferred to a new tube and diluted (100 μl of the supernatant 1:5) and used for further analyses.

After extraction, samples were analysed using enzyme immunoassays (EIA) with a group-specific antibody recognizing 5β,3α,11β-diol glucocorticoid metabolites developed for greylag geese [41] following the protocol by Palme & Möstl [62]. Details of the procedure and cross-reactivities of this assay are published elsewhere [54]. The standard curve ranged from 2 to 500 pg/well and the 50% intercept was about 30pg [54]. For the measurement with EIA, the supernatant was transferred to a TRIS assay buffer and the pH adjusted to 7.5 following [62]. All analyses were run in duplicates; the confidence criterion for the samples was set at a coefficient of variance (CV) of ≤15% for duplicates. The CV for sample duplicates was calculated as the percentage of the standard deviation of the duplicates divided by the mean of the duplicates. This serves as a control of the sample and analysis quality, as we would have re-analysed or excluded samples of CVs > 15 from further analyses. Neither controls nor any samples fell beyond the range of the standards. Concentration limits ranged from 6.22 to 579.16 ng CORTm/g droppings on the control day and 4.46 to 391.63 ng CORTm/g droppings on the challenge day, respectively. Intra- and inter-assay coefficients of variation (CV) were determined from homogenized pool samples and were 2.2 (<15%) and 1.0% (<25%), respectively.

### Determination of diel excretion patterns of corticosterone metabolites under 24 hours of natural light

#### Study population and sample collection

This study was performed in Ny-Ålesund, which is located at the northern side of Brøgger Peninsula at the southern shore of Kongsfjorden, Svalbard (78°55’N, 11°56’E). Here, 24 hours of daylight last from April 18^th^ to August 24^th^. To determine the diel pattern of corticosterone metabolites, we collected data from five 33-day old human-raised goslings. Goslings were collected during peak hatching time from nests of a banded breeding population (see *e*.*g*. [63, 64] for details) and were immediately marked with a small permanent numbered tag in the web of their feet. One human foster parent (M. E. de Jong) provided care for the goslings. Data collection took place from 22:00 on 30^th^ July to 21:59 on 31^st^ July 2013. To be able to collect all droppings from the five individuals (sex and identification band: ♂IA, ♀IC, ♀II, ♂IJ, ♂IX) over 24 hours, samples were collected continuously by changing teams of two, either from above netted, predator-safe cages (length x width x height: 2 x 2 x 1 m) placed on the tundra close to the research station or by following the goslings over the tundra. Samples were frozen within 1 hour after collection at -20°C and ultimately were shipped to the University of Vienna, Department for Behavioural Biology, for analysis. Samples were processed in the same way as described under 2.1.2. In total, we collected 571 dropping samples over the course of 24 hours (IA: 129 samples, IC: 92 samples, II: 101 samples, IJ: 109 samples, IX: 140 samples, S2 Table).

### Statistical analyses

#### Biological validation of the corticosterone enzyme immunoassay

Data sets were analysed by using SigmaPlot 11.0 (Systat Software, San Jose, CA). We tested for normality of the data distribution with Shapiro–Wilk tests (*SW*) and applied appropriate parametric or nonparametric tests whenever applicable.

In order to validate the corticosterone enzyme immunoassay, we split our six-hour sampling period into two three hour periods, based on the maximum gut passage time of barnacle geese. Period 1 (*‘Challenge Period’*), which covered all droppings that resulted from confinement on the challenge day, lasted from 10:00 to 12:59, whereas Period 2 (*‘Recovery Period’*), in which CORTm values should have returned to baseline after the challenge, lasted from 13:00 to 15:59. On both days, we collected a similar number of droppings per individual, *i*.*e*. 3.71 ± 1.98 (mean ± SD, range 1–9) samples per individual on the control day, and 3.13 ± 1.55 (mean ± SD, range 1–7) on the challenge day. As mean values of immunoreactive corticosterone metabolites (Tab. 1) did not differ between males and females either on the control (mean ± SD: males 67.3 ± 30.20, females 69.83 ± 35.86) or challenge day (mean ± SD: males: 86.94 ± 50.36, females 72.92 ± 32.54), we pooled data for further analyses. To test, whether the enzyme immunoassay was sensitive enough to pick up an increase in CORTm on the challenge relative to the control day, we calculated means for each individual during periods 1 and 2 on both days (*n* = 20, Table 1). Whereas data for period 2 were normally distributed (*SW*: *P* = 0.861), data for period 1 were not (*SW*: *P* < 0.05). We opted to present results of Wilcoxon Signed Rank Tests (*WSR*) in both cases for consistency.

**Table 1:** Details of barnacle geese used for the EIA validation.

Individual banding combination	Sex	$\bar{x}$ CORTm Control Period 1 10:00–12:59	$\bar{x}$ CORTm Challenge Period 1 10:00–12:59	$\bar{x}$ CORTm Control Period 2 13:00–15:59	$\bar{x}$ CORTm Challenge Period 2 13:00–15:59
blue—green silver	f	45.27	50.85	20.88	49.79
blue yellow—silver	f	32.41	64.24	36.14	47.30
brown—silver yellow	f	52.34	19.65	111.49	43.43
green green—orange	f	49.82	98.20	14.36	61.45
orange brown—green	f	100.91	213.67	92.26	94.66
red green—green	f	94.07	66.68	151.80	48.98
red green—silver	f	44.19	43.07	139.49	96.01
red silver—orange	f	17.07	53.83	72.52	133.12
yellow—green blue	f	44.71	54.64	77.42	32.64
blue blue—yellow	m	72.40	71.18	27.69	65.21
red—blue silver	m	75.25	107.21	32.92	
red green—white	m		15.41	63.86	23.71
red orange—yellow	m	45.20	65.21		104.58
red silver—white	m		30.59	44.42	
red yellow—brown	m	127.29	396.95	53.81	27.56
red yellow—green	m		110.55	88.78	
silver—red brown	m		50.85	118.51	181.38
silver blue—orange	m		64.24	52.58	42.28
yellow orange—brown	m		19.65	17.38	21.63
yellow red—blue	m	55.70	98.20	108.26	87.28

Banding combinations (right leg–left leg) and sex (f = female, m = male) of 20 captive barnacle geese housed in the animal care facility at the University of Groningen, Netherlands), from which dropping samples were collected. Means per individual of immunoreactive corticosterone metabolites (ng CORTm/g droppings) on control and challenge day split into periods 1 and 2 are given. Empty cells indicate that no dropping samples could be collected from that individual in the given period.

#### Determination of diel excretion patterns of corticosterone metabolites under 24 hours of natural light

Individual differences of defecation rates between the five hand-raised goslings were analyzed with Kruskal-Wallis Analyses of Variance by Ranks (*KW ANOVAs)*. We performed post-hoc tests applying Dunn’s method (*Dunn)* for multiple comparisons of ANOVAs by ranks with unequal samples sizes.

To detect a potential diurnal pattern of corticosterone metabolite excretion under 24 hours of natural light, data were analysed using CircWave software V. 1.4 (Courtesy of R. Hut, GELIFES, University of Groningen). CircWave uses harmonic linear regression to fit a sinusoidal curve to the data and to test its significance against a fitted horizontal line using an F-test. We evaluated the raw data by fitting the following function f(t) = c + a **·** sin ($\frac{2\pi t}{24}$) + b **·** cos ($\frac{2\pi t}{24}$) to each individual after transferring raw time into decimal time. From the results of CircWave we calculated the polar coordinates (*r*, θ) to display all five individuals at peak time on a polar plot. Vector length *r* was calculated using the following equations: *X* = (sin (θ) Ind. 1 + sin (θ) Ind. 2 + sin (θ) Ind. 3 + sin (θ) Ind. 4 + sin (θ) Ind. 5; *Y* = (cos (θ) Ind. 1 +(cos (θ) Ind. 2 +(cos (θ) Ind. 3 +(cos (θ) Ind. 4 +(cos (θ) Ind. 5; *r* = $\sqrt{X^{2}+Y^{2}}$. Finally, we tested for non-uniformity of the calculated angles with the Rayleigh z test for data in a circular distribution by calculating: z = n · *r*^2^. Prior visual inspection of the calculated angles revealed no sign of bimodality of the data around the 24 hour the circle and thus indicated that the application of the Rayleigh z test was appropriate.

All statistical results are given two-tailed with an α of 0.05.

## Results

### Biological validation of the corticosterone enzyme immunoassay

**Fig 1:**
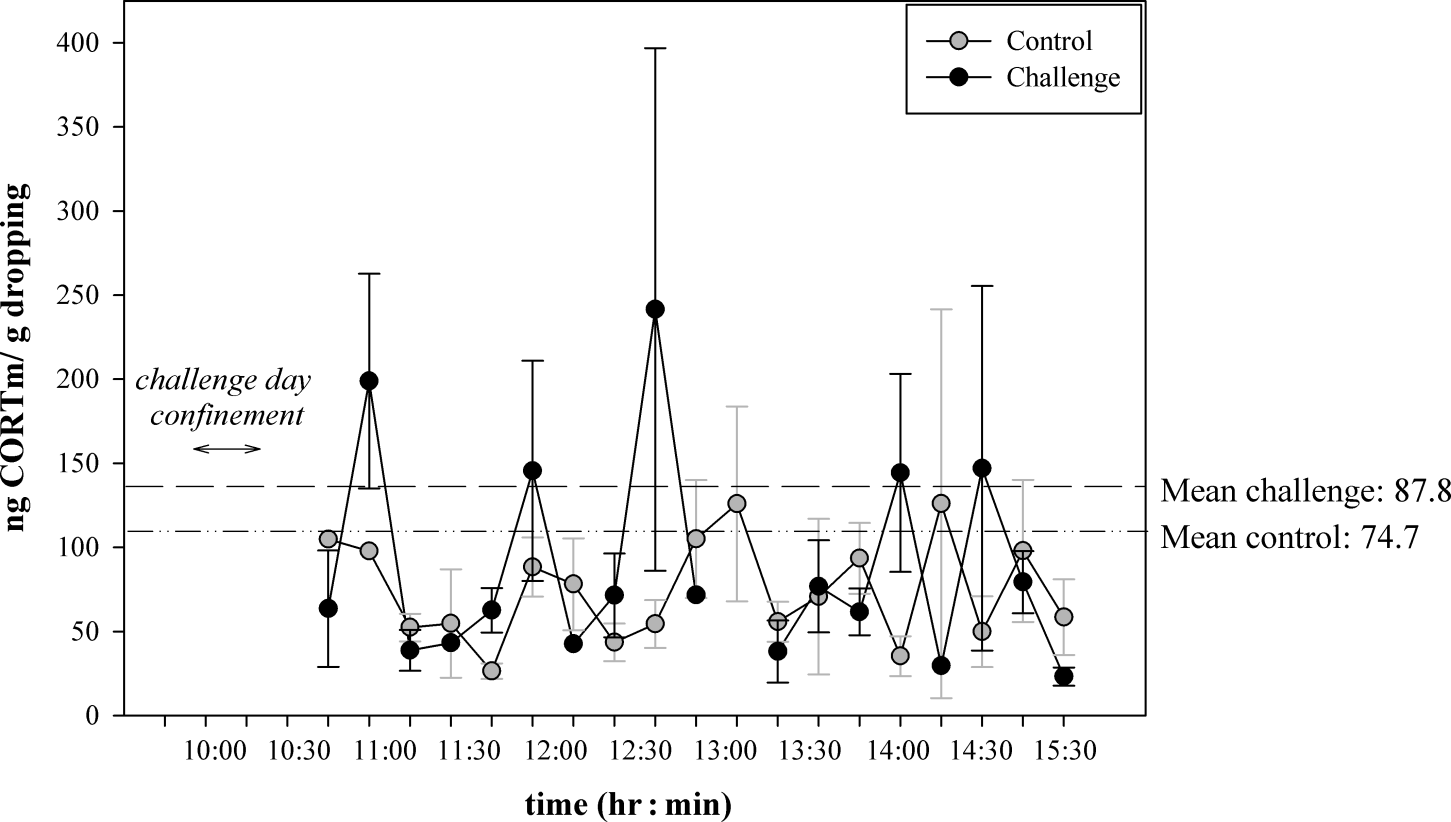
Excretion profiles of immunoreactive corticosterone metabolites in ng CORTm/g dropping on control (grey circles) and challenge (black circles) day over time (GMT +1 hr.). Each point represents the mean (± S.E.M.) of all samples collected during a 15-min interval, *e*.*g*. the first data point represents the mean of dropping samples collected between 10:45–10:59. Horizontal lines indicate the overall mean during control (line style: dash–dot–dot) and challenge (line style: dash) day. Duration of confinement on the challenge day is shown by ←→.

The excretion profiles of CORTm on the control and challenge day are presented in Fig 1. Visual inspection revealed a difference in temporal excretion patterns on the control and challenge day. Relative to the control day, two pronounced peaks occurred on the challenge day during the time intervals from 11:00–11:14 and 12:45–12:59, respectively. Although in birds, renal and faecal matter are jointly excreted via the cloaca [50], the turn-over time of corticosterone in uric acid is faster than from faecal matter, suggesting that the first peak (11:00–11:15) reflects corticosterone metabolites from uric acid, and the second peak (12:30–12:45) might be a sign of corticosterone metabolites from faecal excretion. This is consistent with data found in other goose and sheldgoose species [56, 65, 66].

During the first three hours (Challenge Period), individual mean CORTm was higher on the challenge day relative to the control day (*WSR*: *Z* = -2.166, *P* = 0.033, Table 1). This was not the case in the three hours of the Recovery Period (*WSR*: *Z* = -0.362, *P* = 0.744, Table 1). This indicates that the confinement proved to be a valid stressor for the geese and that the assay was sensitive enough to pick up the corticosterone response. We, therefore, chose to analyse further dropping samples with this assay.

### Determination of diel excretion patterns of corticosterone metabolites under 24 hours of natural light

Mean defecation rate of the five individuals over the course of 24 hours was 4.75 droppings per hour (range: 3.80–5.80 droppings). CORTm means per time interval differed between individuals (*KW ANOVA*: *H* = 25.945, *df* = 4, *P* < 0.001): Individual IC had higher baseline CORTm values than individual IA (*Dunn*: *Q* = 2.901, *P* < 0.05), and individual IJ (*Q* = 4.881, *P* < 0.05), respectively. Additionally, individual II had higher CORTm values than individual IJ (*Q* = 3.410, *P* < 0.05; all other pairwise multiple comparisons ns).

**Fig 2:**
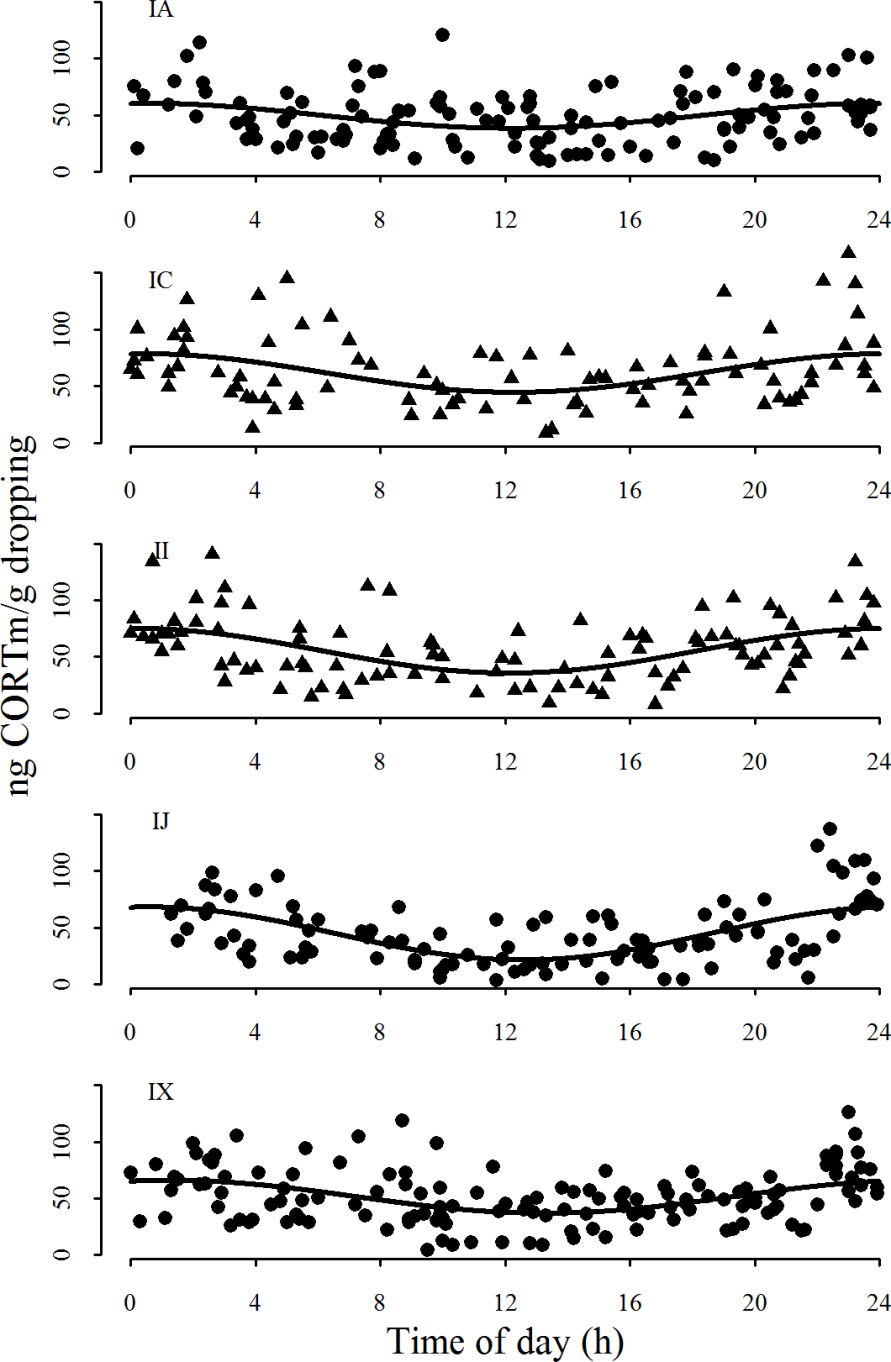
Individual CORTm excretion patterns in ng CORTm/ g dropping of the five human-raised barnacle goslings (♂IA, ♀IC, ♀II, ♂IJ, ♂IX) over 24 hours. Males are depicted in circles, females in triangles. Model lines for each individual were calculated using the CircWave time function: f(t) = c + a **·** sin ($\frac{2\pi t}{24}$) + b **·** cos ($\frac{2\pi t}{24}$). Values c, a and b for all individuals are listed in Table 2.

**Fig 3:**
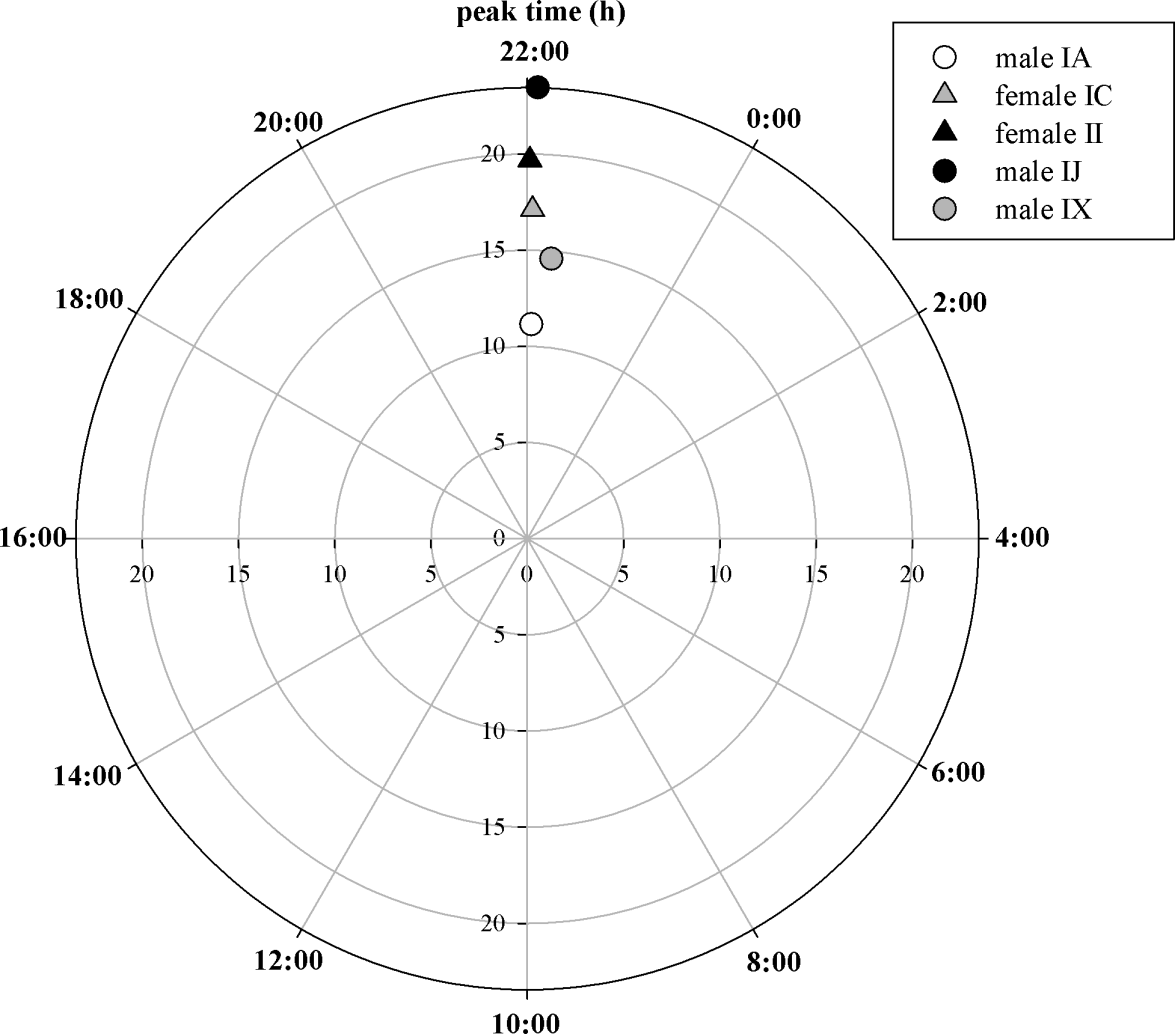
Polar locations of time of day (in hours) when CORTm peaks. This shows that the CORTm peak of all five individuals occurs at approximately the same time. Radial values display the vector length r, the radial distance from the origin, with larger values being indicative of a stronger diel pattern (for calculations see Table 2). Collection started at 22:00 (July 30^th^, 2013) and lasted until 21:59 (July, 31^st^, 2013).

Each individual showed a highly significant rhythmic pattern (Fig 2, Table 2). These rhythmic patterns were not random among individuals and showed highest levels of CORTm around 22:00. This non-uniform distribution was statistically supported (Rayleigh test: *Z*_4.970_, *P* < 0.0001, n = 5, Fig 3) and indicates a rhythmic pattern of CORTm excretion under 24 hours of natural daylight in Arctic barnacle geese.

**Table 2:** Individual CircWave results.

Individual	c	a	b	p	R^2^_*unadj*._	*tan* (θ)	peak time (h)	r
**IA**	49.45	0.615	11.12	0.001	0.10	0.0553	0.2110	11.1370
**IC**	61.35	1.25	17.05	<0.001	0.15	0.0733	0.2795	17.0958
**II**	55.15	0.68	19.66	<0.001	0.22	0.0359	0.1321	19.6718
**IJ**	44.67	3.38	23.19	<0.001	0.35	0.1458	0.5528	23.4350
**IX**	51.25	4.67	13.77	<0.001	0.18	0.3391	1.2489	14.5404

Results of the CircWave analyses solving the following time function

f(t) = c + a · sin ($\frac{2\pi t}{24}$) + b · cos ($\frac{2\pi t}{24}$) per individual (IA, IC, II, IJ, IX). All individuals showed a highly significant diel pattern of CORTm excretion (p ≤ 0.001). The coefficient of determination (R^2^_unadjusted_) is also presented. From the CircWave results, we calculated the tangent of the polar angle (a/b = *tan* (θ), peak time (arc tangent in rad converted into decimal time in hours) and radius r (√ of a^2^+b^2^) for each individual.

## Discussion

In this study we present the first complete diel CORTm excretion pattern in barnacle goslings living under 24 hours of continuous natural light. Contrary to our hypothesis, we found that corticosterone metabolites from droppings showed both a clear peak and nadir that is suggestive of a circadian rhythmic pattern. The maximum peak occurred at the start of sample collection around 22:00, which corresponds to highest levels in plasma between 19:00–21:00. Whereas most studies fail to find a rhythmic pattern of glucocorticoid excretion under constant daylight [7, 9], we are aware of one study in humans, where a circadian pattern of cortisol was maintained in a period of 24 hours of daylight (April/May) in male construction workers on Svalbard [67]. Our unexpected finding raises the question of why geese showed a rhythmicity in corticosterone excretion.

### Activity patterns of geese, humans and predators

Daily variation in baseline glucocorticoid levels is believed to play a large role in regulating metabolism of an animal by promoting feeding in order to regulate the deposition and storage of energy [14, 68]. As do other species of geese, barnacle geese usually feed during the day and rest at night in lower latitudes [69–71]. In our study area, where 24-hours of polar daylight last from April 18^th^ to August 24^th^, geese use the night hours for feeding too, but engage in prolonged resting bouts around midnight in August (M. J. J. E. Loonen, pers. obs.). One possible explanation, therefore, is that the peak in CORTm we observed at the end of July might incite geese for an extended feeding bout before a prolonged resting period around midnight.

Furthermore, in the present study, human activities in Ny-Ålesund were considerably reduced during night time in the summer months, which might increase predation risk around town at night [72] due to higher encounter rates of polar foxes (*Vulpes lagopus*) and polar bears (*Ursus maritimus*) in close vicinity to town during the quieter hours (M. E. de Jong, M. J. J. E. Loonen, I. B. R. Scheiber, B. M. Weiß, per obs.). The human-raised goslings in this study might have been more vigilant at night due to a general prevalence of predators in the night hours, or because they were affected by increased vigilance from their wild conspecifics, which, in turn, may have affected their CORTm excretion pattern.

Another feasible explanation is that the observed peak in CORTm in the late evening hours is not indicative of diel rhythmicity but occurred in response to some unknown disturbance in the hours prior to the start of our data collection. What argues against this idea is that CORTm decreased gradually over six hours rather than suddenly, which would have been expected if the peak had been caused by a disturbance. Furthermore, the peak value of CORTm at 22:00 is well below the peak values collected in response to an acute stressor, *i*.*e*. the confinement challenge in the validation experiment. We can also exclude the possibility that the higher values were caused by our presence, as we then would have expected the peak to occur anywhere between 23:00 and 01:00.

### Possible mechanisms involved in maintaining rhythmicity

Relative to geese at lower latitudes, which show a peak in corticosterone levels right around waking, there seems to be a shift of the diel pattern in CORTm excretion in the barnacle goslings in Svalbard. Although the light-dark cycle, *per se*, is absent in the Arctic during the month of July, several circadian systems are fully expressed even under 24 hours of continuous light, at least in migratory animals [19, 21, 22, 73]. This rhythmicity might be triggered, as has been recently suggested, *e*.*g*. by changes in light intensity throughout the day [30], ambient temperature [21] and/ or by melatonin as the essential product of circadian master clocks [19, 22]. The limited number of investigations on migratory polar birds that assessed melatonin profiles under 24-hours of natural light found a diel rhythm of melatonin in plasma [19, 21, 22, 29]. Interestingly, the timing of the circulating melatonin profile in Lapland longspurs (*Calcarius lapponicus)* [21] overlapped agreeably with that of our CORTm excretion patterns in barnacle goslings. In resident Svalbard ptarmigan, however, a melatonin rhythmicity is not expressed from May to July, whereas plasma melatonin levels vary throughout the day in all other months of the year [30]. As our data collection took place at the very end of July, goslings may have just started to entrain their corticosterone excretion pattern by responding to an emerging melatonin rhythmicity triggered by subtle Zeitgebers, such as light intensity or ambient temperature. The pattern generally expected in geese, *i*.*e*. an early corticosterone morning peak, might only emerge later on, once photoperiod as an environmental cue becomes dependable again.

Unfortunately, nothing is known about melatonin expression in geese in the Arctic. A prospective line for further investigations is the measurement of melatonin from droppings as, so far, it has been measured exclusively from plasma. Melatonin, however, has already been successfully measured non-invasively in fish from holding tank water as well as from mouse urine and human saliva [74–76]. Quantifying both corticosterone and melatonin metabolites simultaneously from the same faecal samples at different time points over the polar summer (*i*.*e*. at the onset, over the course of, and towards the end of the 24-hour light period) might shed light on the mechanisms involved in hormonal and behavioural rhythmicity as it relates to variables such as light intensity, temperature, etc.

### Effects of age

Very few studies have examined diel corticosterone rhythms in young birds [16, 77–79], which showed that adult-like diel rhythmicity patterns may be observed shortly before sexual maturity in domestic chickens (*Gallus gallus domesticus*, [78]) and shortly before fledging in western screech-owls (*Otus kenicottii*, [77]), thin-billed prions (*Pachyptila belcheri*, [16]) and white-crowned sparrows (*Zonotrichia leucophrys nuttalli*, [79]). The only study that explicitly examined when a fully functional avian corticosterone rhythm develops was performed in domestic fowl in the laboratory under specified light conditions, *i*.*e*. 14L:10D [78]. Chicks developed the first, phase-shifted rhythmicity between five and eleven weeks of age, with a corticosterone increase during or just prior to the onset of darkness. The characteristic adult corticosterone pattern was only apparent once chicks were approximately 17 weeks old, *i*.*e*. around sexual maturation. This is similar to rats kept at 12L:12D, in which a rhythm first appeared at three weeks of age, but an adult pattern was only obtained when pups were five weeks old [80]. Hence, the phase-shifted rhythm we observed in barnacle goslings could similarly have been a first, pre-mature rhythmicity subject to change in older individuals.

### Methodological assessment: Sampling regime, sample size

The main goal of this study was to describe the excretion pattern of basal CORTm over a complete polar day in barnacle goslings. As the detection of short steroid metabolite peaks requires frequent sampling [35, 56], we opted to collect *all* samples, rather than at specific times points, a methodology that was suggested as the best option for describing a diel pattern [33]. This is a logistically difficult collection scheme, and so far samples have never been collected in this detailed manner over 24 hours outside the laboratory. Utilising human-raised goslings allowed us to perform the fine-tuned data collection we were aiming for but limited the number of individuals and the number of 24hr cycles that could be sampled without excessive manpower. Accordingly, it might be argued that one day only might not be enough to describe the observed pattern as being a diel rhythm, as it may have been caused by something unanticipated. Although this is a valid claim it was a necessary trade-off between sampling accuracy and length of the sampling period, and goslings showed no aberrant behaviour indicative of stress to the familiar humans who performed the sample collection. As we are well aware that multiple 24hr periods (possibly derived from several studies) would need to be measured in order to assess if the observed patterns represent a true diel rhythm, we opted to refer to a diel pattern throughout, rather than calling it a rhythm.

## Conclusions

Juvenile barnacle goslings showed a circadian pattern in CORTm excretion under 24h of natural daylight, which deviated from the CORT patterns of barnacle geese at lower latitudes by peaking in the evening rather than the morning hours. Possible explanations for the observed pattern include the young age of the study subjects as well as human and predator activity patterns in the research area, which should be addressed in future studies. Measuring CORTm from faeces further proved to be a suitable alternative to blood sampling for evaluating corticosterone profiles under continuous daylight. A promising avenue for future research comprises also measuring melatonin from faeces to better understand the mechanisms of circadian rhythms under continuous light regimes.

## Supporting information

S1 Table Validation CORTm by individuals on control and challenge day. (XLSX)

S2 Table Diurnal pattern CORTm by individuals. (XLSX)
